# Supplementary figures and images for: Improvement in neoantigen prediction via integration of RNA sequencing data for variant calling
Source: Front Immunol. 2023 Sep 4;14:1251603. doi: 10.3389/fimmu.2023.1251603 (PMC10507271; doi:10.3389/fimmu.2023.1251603)

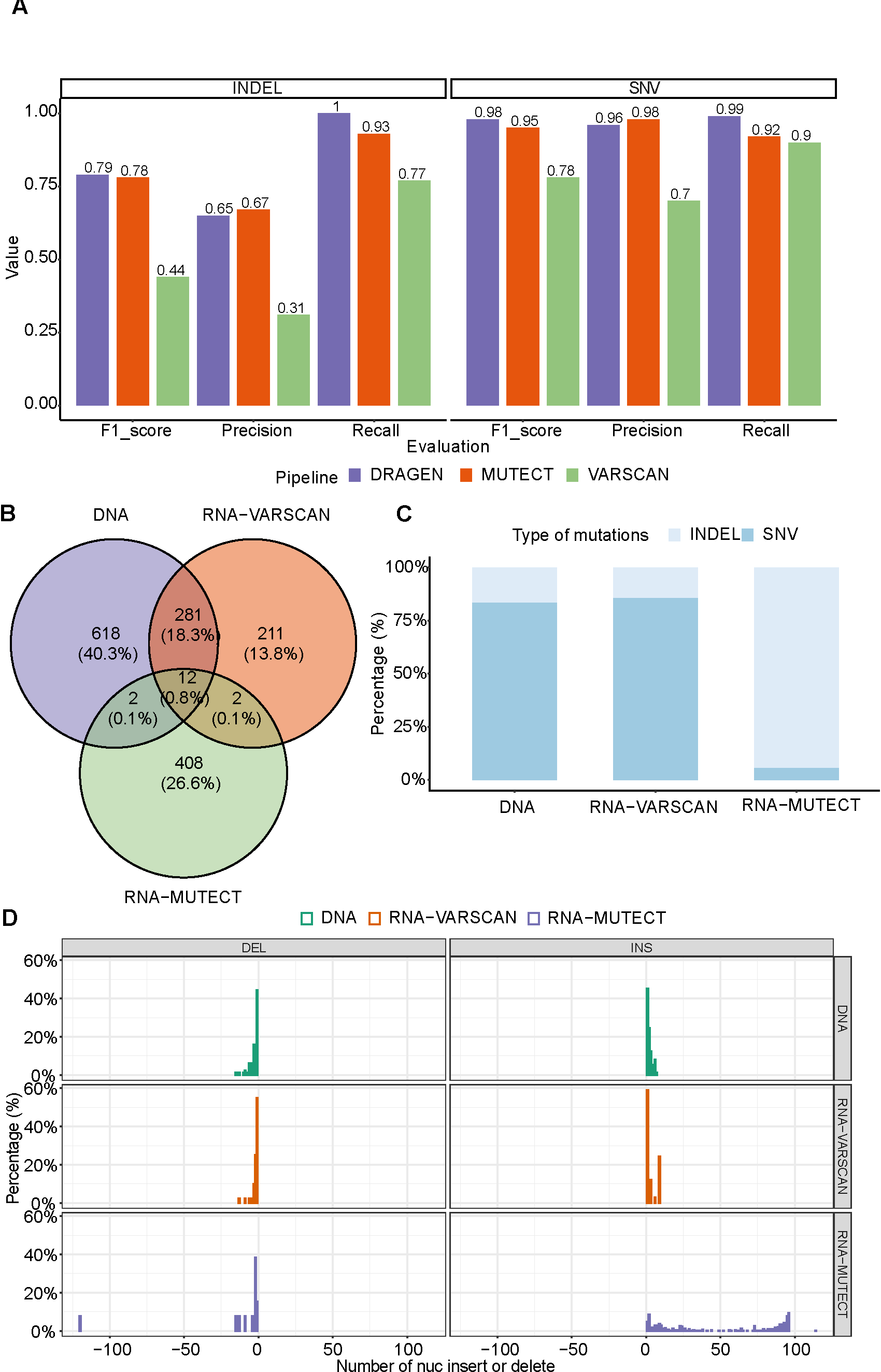

Supplement: Supplementary Figure 1 — Evaluation of mutation calling tools for DNAseq and RNAseq data (A) Comparison of performance of three indicated mutation callers on a reference DNAseq dataset. (B) A Venn diagram illustrates the number of mutations identified by Dragen and two RNA mutation callers, VarScan and MuTect2. (C) Proportions of SNV and indel mutations called by indicated tools. (D) Length distribution of INDEL mutations called by indicated tools [file Image_1.tiff]

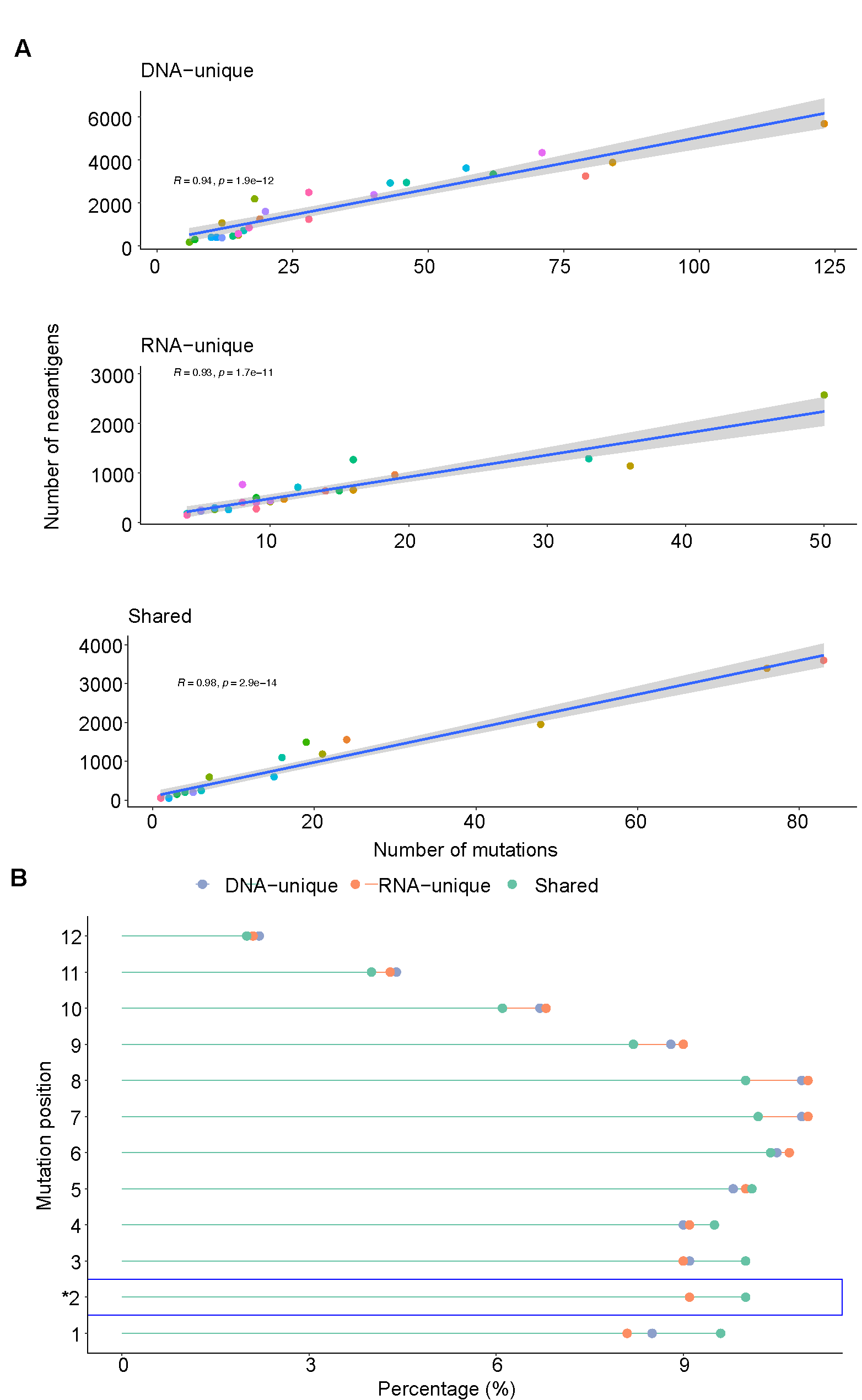

Supplement: Supplementary Figure 2 — Distribution of mutation positions of DNAseq and RNAseq derived neoantigen (A) Correlation between the numbers of variants and neoantigens within the indicated groups. (B) A lollipop plot displays the percentage of neoantigens from the indicated groups that contain mutations at positions 1 to 12. The blue box represents the anchor site of the peptide and HLA-I molecule. [file Image_2.tiff]

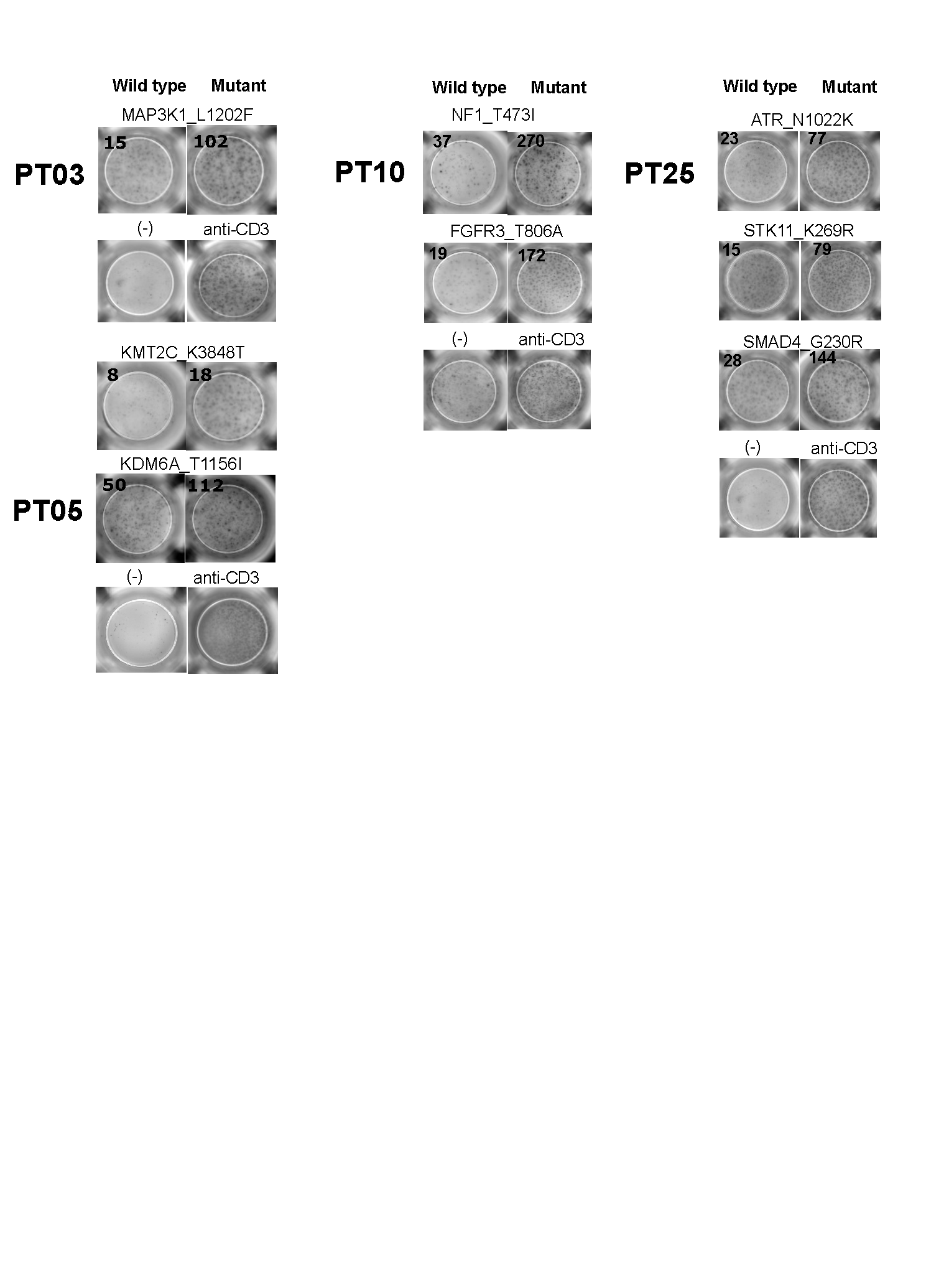

Supplement: Supplementary Figure 3 — ELISpot assays on eight long peptides which result in 2-fold change of IFN-γ spots. [file Image_3.tiff]

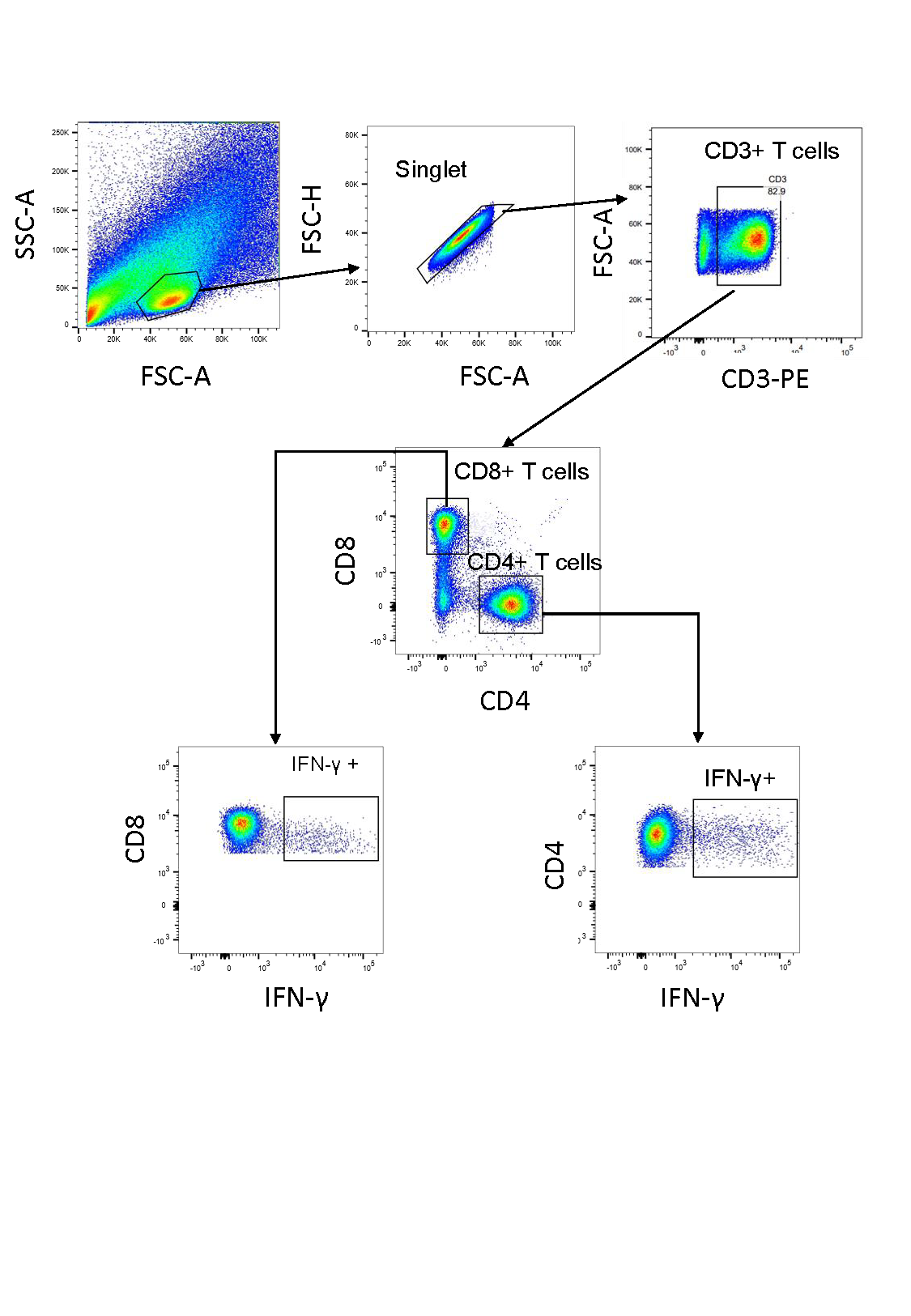

Supplement: Supplementary Figure 4 — Gating strategy for detecting IFN-γ production from CD4+ and CD8+ T cells in LP-stimulated PBMCs of 4 CRC patients. [file Image_4.tiff]
